# Supplementary figures and images for: Calcium phosphate formation and deposition in ischemic neurons
Source: PLoS One. 2025 Jan 16;20(1):e0317055. doi: 10.1371/journal.pone.0317055 (PMC11737781; doi:10.1371/journal.pone.0317055)

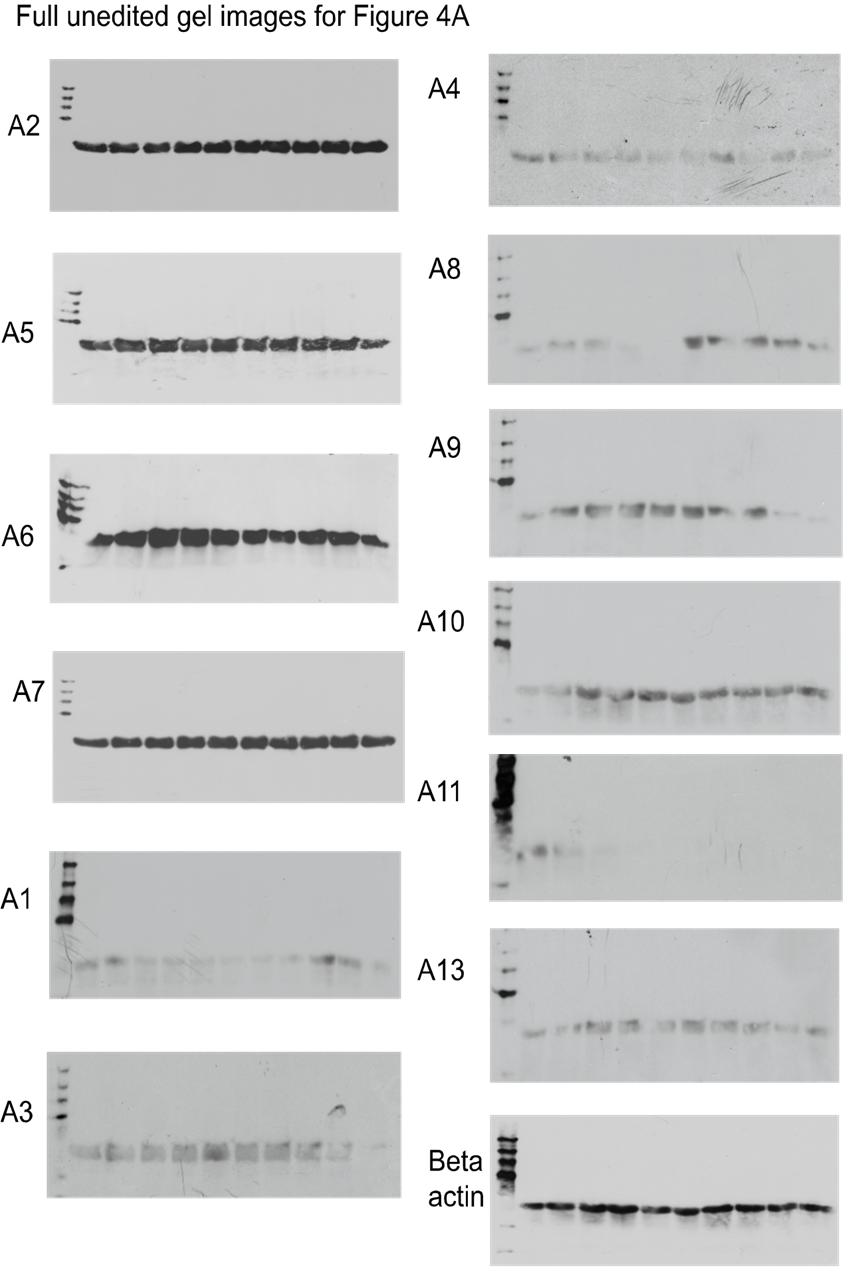


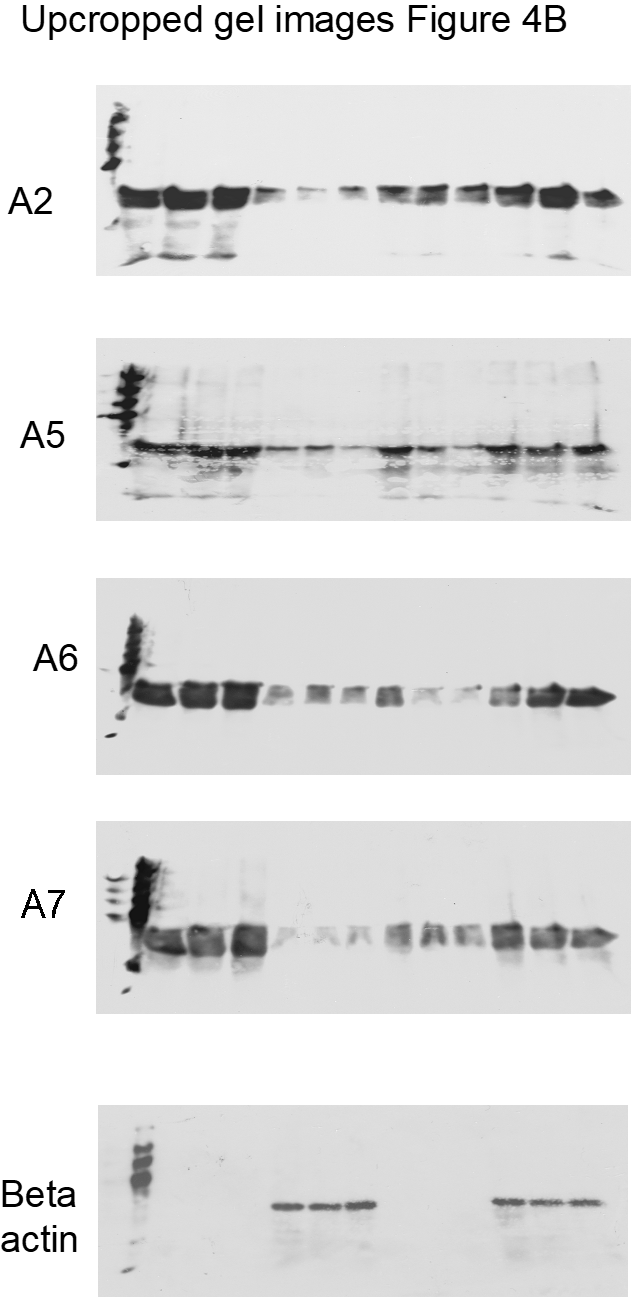


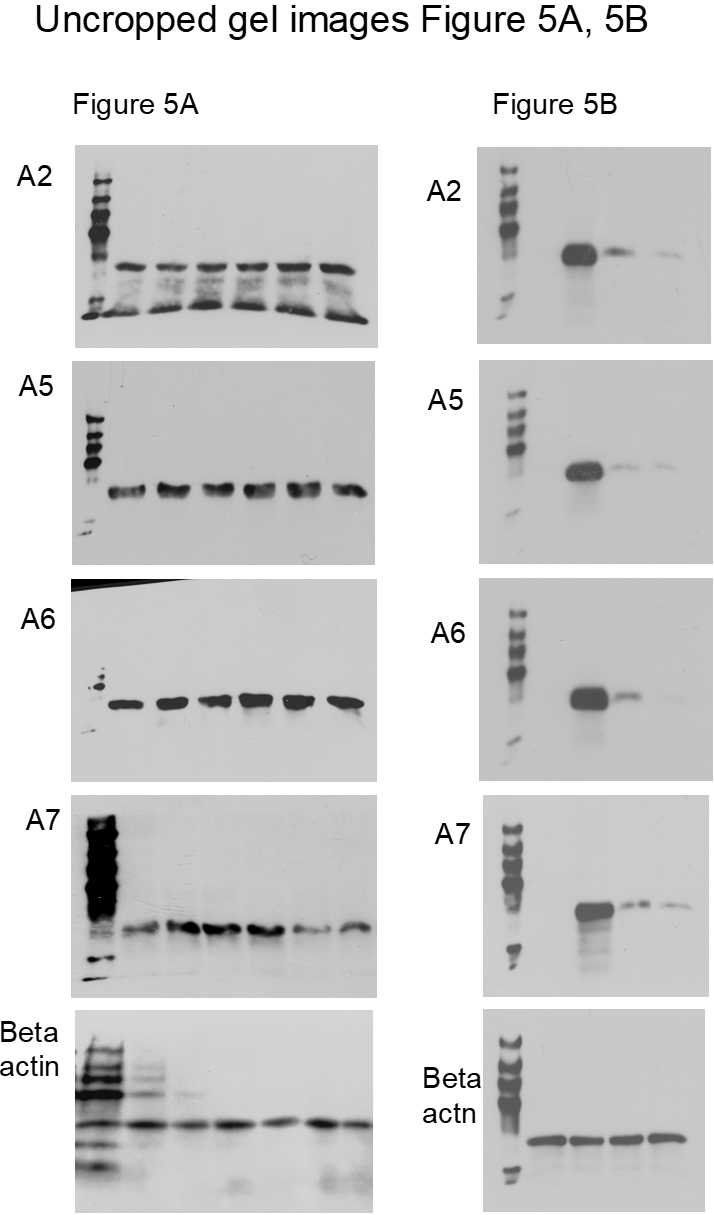


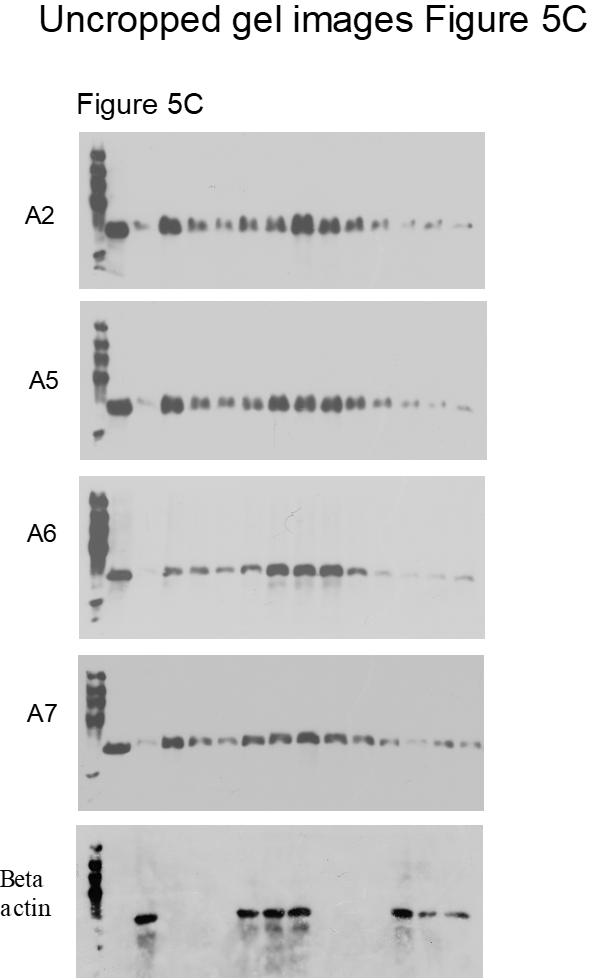


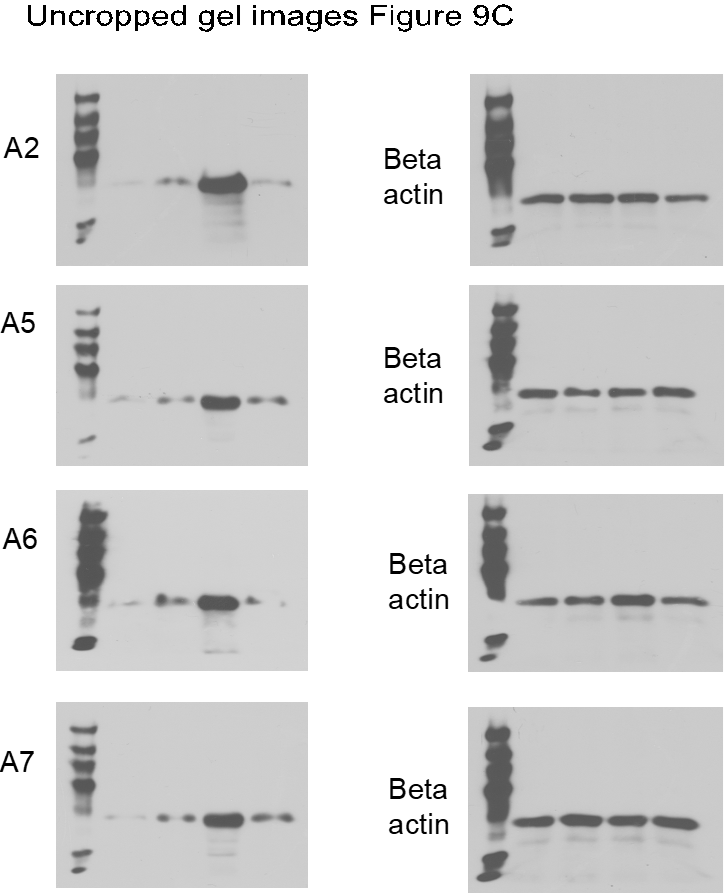


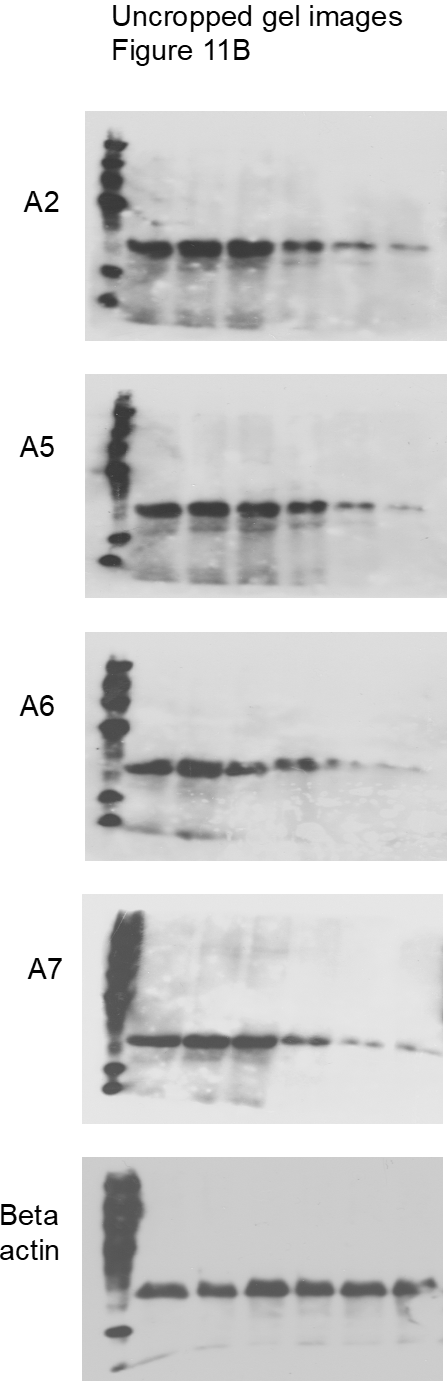

Supplement: S1 File — (DOCX) [file pone.0317055.s002.docx]
