# Supplementary material for: Calcium phosphate formation and deposition in ischemic neurons
Source: PLoS One. 2025 Jan 16;20(1):e0317055. doi: 10.1371/journal.pone.0317055 (PMC11737781; doi:10.1371/journal.pone.0317055)
Supplement: Attachment — Submitted filename: 2024 Response to Reviewer 2.pdf [file pone.0317055.s004.pdf]

Response to Reviewer 2

The authors would like to thank the reviewer for providing insightful comments. The manuscript has been revised based on the reviewer's concerns. All changes are marked in blue in the revised manuscript. The reviewer's concerns are addressed here.

Reviewer's concern: My concerns were not sufficiently addressed by the response of the authors.

> **Response**: Sorry that the authors did not address fully the reviewer's concerns. In this revision, these concerns have been carefully addressed and substantial changes have been made accordingly.

Reviewer's concern: - Selecting areas for quantification in Fig 3 appears very arbitrary given the high background.

> **Response**: Figure 3A, B and associated data have been removed from the revised manuscript. Results from the remaining p-nitrophenyl phosphate (pNPP) test would be sufficient to support the observation that the activity of alkaline phosphatase was increased in the cortex in response to ischemic stroke.

Reviewer's concern: - I do not see, why quantification for Fig 6 distribution of actin and annexins poses a major problem.

> **Response**: The distribution of annexins in reference to that of β actin filaments has been measured and presented in Fig. 6B.

Reviewer's concern: - Prevention of CaP deposition for treatment as proposed in the discussion would be too late as the cell membranes are already damaged. The response to this criticism does not settle the point.

> **Response**: The authors are in agreement with the reviewer. The statement on "prevention of CaP deposition for the treatment of myocardial infarction" has been removed from the Abstract and Discussion sections. The revised manuscript focusses primarily on the molecular mechanisms of calcium phosphate formation and deposition in ischemic neurons.

Reviewer's concern: - The primary data were now supplied as supplement.

> **Response**: Thanks.
