# Supplementary material for: Calcium phosphate formation and deposition in ischemic neurons
Source: PLoS One. 2025 Jan 16;20(1):e0317055. doi: 10.1371/journal.pone.0317055 (PMC11737781; doi:10.1371/journal.pone.0317055)
Supplement: Attachment — Submitted filename: 2024 Response to Reviewers PlosOne.docx [file pone.0317055.s003.docx]

**Response to the Reviewers**

The authors would like to thank the reviewers for providing thoughtful and informative comments to the manuscript. The manuscript has been revised in accordance to the reviewers’ comments. Please note that all revisions are marked in blue in the Word document. The comments are addressed here.

**Response to Reviewer 1**

The manuscript entitled “Calcium phosphate formation and deposition in ischemic neurons”, by Shi Q. Liu, John B Troy, Jeremy B Goldman and Roger J. Guillory, describes a potential mechanism for the formation of calcium phosphate precipitates in cortical neurons post-ischemia. This manuscript proposes a compelling mechanism where several annexins form complexes with CaP, in a cytoskeletal dependent manner. However, the argument the author makes would be strengthened by providing more evidence to support and clarify the logic of their proposed mechanism. Some key assumptions require further justification and /or clarification.

Concerns:

1) In the current literature intraneuronal CaP precipitates are considered a post-ischemic protective step of calcium homeostasis. How do the authors reconcile this view with their conclusion that preventing CaP deposition during ischemia can be protective and a potential treatment strategy, particularly while the plasma membrane integrity is still compromised (see Figure 13 C)?

**Response:** Intracellular CaP precipitation may represent a protective mechanism against Ca^2+^ overload and its cytotoxicity in ischemic neurons [*Ramonet D, Pugliese M, Rodríguez MJ, de Yebra L, Andrade C, Adroer R, Ribalta T, Mascort J, Mahy N. Calcium precipitation in acute and chronic brain diseases. J Physiol Paris. 96(3-4):307-312, 2002.*]. However, a consensus from a larger number of previous investigations is that the presence of intracellular CaP nanoparticles can cause cell injury and death. For instance, the presence of CaP nanoparticles in human vascular smooth muscle cells can cause cell death [*Ewence AE, Bootman M, Roderick HL, Skepper JN, McCarthy G, Epple M, Neumann M, Shanahan CM, Proudfoot D. Calcium phosphate crystals induce cell death in human vascular smooth muscle cells: a potential mechanism in atherosclerotic plaque destabilization. Circ Res. 103(5):e28-34, 2008*]. Other studies support this observation [*Epple M. Review of potential health risks associated with nanoscopic calcium phosphate. Acta Biomaterialia 77, 1-14, 2018; Z.L. Shi, X. Huang, Y.R. Cai, R.K. Tang, D.S. Yang. Size effect of hydroxyapatite nanoparticles on proliferation and apoptosis of osteoblast-like cells. Acta Biomater., 5 (2009), pp. 338-345; Z. Xu, C. Liu, J. Wei, J. Sun. Effects of four types of hydroxyapatite nanoparticles with different nanocrystal morphologies and sizes on apoptosis in rat osteoblasts. J. Appl. Toxicol., 32 (2012), pp. 429-435; H. Turkez, M.I. Yousef, E. Sonmez, B. Togar, F. Bakan, P. Sozio, A.D. Stefano. Evaluation of cytotoxic, oxidative stress and genotoxic responses of hydroxyapatite nanoparticles on human blood cells. J. Appl. Toxicol., 34 (2014), 373-379; E. Sonmez, I. Cacciatore, F. Bakan, H. Turkez, Y.I. Mohtar, B. Togar, A.D. Stefano. Toxicity assessment of hydroxyapatite nanoparticles in rat liver cell model in vitro. Hum. Exp. Toxicol., 35 (2016), 1073-1083; Y. Jin, X.L. Liu, H.F. Liu, S.Z. Chen, C.Y. Gao, K. Ge, C.M. Zhang, J.C. Zhang. Oxidative stress-induced apoptosis of osteoblastic MC3T3-E1 cells by hydroxyapatite nanoparticles through lysosomal and mitochondrial pathways. RSC Adv., 7 (2017), 13010-13018; Y. Dautova, D. Kozlova, J.N. Skepper, M. Epple, M.D. Bootman, D. Proudfoot. Fetuin-A and albumin alter cytotoxic effects of calcium phosphate nanoparticles on human vascular smooth muscle cells. PLoS One, 9 (2014), e97565*]. Based on these observations, it is possible that the impact of CaP formation on cell survivability may depend on the level of free CaP nanoparticles and the degree of CaP deposition to intracellular structures. The presence of a low density of free CaP nanoparticles without significant deposition to intracellular structures may be protective against Ca^2+^ overload and cytotoxicity; whereas the deposition of a high level of CaP nanoparticles to intracellular structures, especially those critical to cell signaling and integrity, may elicit a harmful impact on cell survivability.

β actin filaments support many neuronal functions such as signal transduction, maintenance of cell integrity, regulation of synaptic plasticity, and control of axonal branching and regeneration. In particular, the dynamic assembly and disassembly processes of β actin is essential to neuronal survival [*Patrinostro X, Roy P, Lindsay A, Perrin BJ. Essential nucleotide- and protein-dependent functions of Actb/β-actin. PNAS 115 (31) 7973-7978, 2018*]. Exposure to phalloidin, a substance that stabilizes actin filaments and prevents the dynamic actin processes, can cause cell death [*Cooper, J. A. Effects of cytochalasin and phalloidin on actin. J. Cell. Biol. 105, 1473–1478, 1987*]. CaP deposition on β actin filaments, as observed in this investigation, can interfere with these dynamic actin processes, thereby contributing to neuronal injury and death. The points above are addressed in the revised manuscript (page 19 – 20 of the Word document).

2) Using a biochemistry approach the authors conclude that annexins translocate from the cell membrane to the cytoskeleton. However, they also conclude that when the translocation occurs the plasma membrane integrity is compromised. A leaky plasma membrane is the first step versus irreversible cell death and disassembly of the cytoskeleton. Indeed, recently a paper has shown that ischemic cortical neurons display a dramatic increase in their F-actin content in the somatodendritic region, and that this response is inversely correlated to plasma membrane leakage. Can the authors support their conclusion by using an intracellular in situ approach to monitor the interaction between Annexins and the cellular cytoskeleton post-ischemia? For example PLA?

**Response:** In this investigation, an in situ approach was used to demonstrate the interaction between annexins and β actin filaments (Fig. 8 in the manuscript). Freshly prepared healthy mouse brain specimens, instead of ischemic brain specimens, were used for this test. The reason is that annexin – β actin filament interaction occurs in ischemia, making it difficult to distinguish between the newly induced annexin – β actin filament interaction and the ischemia-induced annexin – β actin filament interaction.

In this test, individual annexins were added to brain sections in the presence of Ca^2+^ and PO_4_^3-^, in the presence or absence of an annexin, in the presence or absence of monomeric β actin (capable of binding to annexins to block annexin – β actin filament interaction), and in the presence or absence of phalloidin. The outcomes can be used to evaluate whether annexins bind to β actin filaments to cause CaP deposition to β actin filaments as well as whether the phalloidin-induced stabilization of β actin filaments interferes with annexin binding to the β actin filaments and CaP deposition.

Please note that the total level of β actin was not changed from sham control to ischemic stroke as evaluated by immunoblot analysis in this investigation (Figure 4 in the manuscript).

3) In the manuscript is not explained why when the annexins are added to the healthy mouse brains, this resulted in cerebral CaP deposition and Anx binding to the cytoskeleton. Why in these healthy brains the exogenously added Anx wouldn’t just remain bound to the plasma membrane? Why this manipulation would even cause infarction?

**Response:** The relative level of annexin binding to the cell membrane or β actin filaments may depend on the quantity of exogenous annexins delivered to cells. At a large quantity, the annexin binding sites on the inner face of the cell membrane may all be bound with annexins, and the excessive exogenous free annexins may bind to β actin filaments. Free annexins within the cell can attract calcium ions, which in turn attract phosphate ions to form CaP nanostructures on one side of a disk-like annexin molecule. The other side of the annexin molecule can bind to β actin filaments, resulting in annexin/CaP complex deposition onto the β actin filaments or β actin filament calcification. This process contributes to neuronal death and brain infarction. These points are addressed in the revised manuscript (page 20 of the Word document).

4) In Figure 6 the green actin signal is expected to have the same intensity and distribution in all images, but it doesn’t. Why?

**Response:** This could be a result of variations in experimental preparations and locations for specimen collection among different mice.

5) Figure 11 is the most convincing piece of evidence that annexins are connected to calcium precipitates and infarct size. However, these findings are counterintuitive based on the current knowledge that knocking down annexins would result in plasma membrane damage and excessive calcium influx/levels.

**Response:** It is possible that knocking down annexins could result in plasma membrane damage and excessive calcium influx. Even though additional injury may be induced by annexin siRNA delivery, silencing of the annexin genes resulted in a significant alleviation of CaP deposition and brain infarction, supporting the role of annexins in ischemic neuron calcification and infarction.

6) The authors should make sure that all citated papers are correctly referenced. For example Liu 2019 cited on page 28 does not have any mention of CaP depositions.

**Response:** This error has been corrected. It should be reference #6.

Conclusion: Overall, this manuscript represents an informative new set of experiments that provides insights in the connection between Annexins and CaP precipitates. However, by addressing the limitations outlined above, the authors could strengthen the overall impact of their work.

**Response:** Thanks. The comments have been addressed to our best knowledge.

**Response to Reviewer 2**

Reviewer #2: This study aims to establish that CaP deposition occurs early after an ischemic stroke and that it participates in the associated injury. The authors propose a mechanism after which specific annexins are crucial for CaP deposition via binding to ß actin filaments.

First, the study tries to establish the presence of CaP deposits after ischemia in a mouse model system. Then the expression and subcelluar distribution of annexins is investigated by Western blot followed by an analysis of binding of annexins to ß actin. The alteration in distribution of actin and annexins was investigated by immunofluorescence after stroke induction. Furthermore, actin and annexin dependent CaP deposition was investigated. As a behavioral measure, grip strength was investigated and compared under the different conditions for the lesioned and intact site. Finally, the specific effect of particular annexins was investigated by blocking annexin expression by siRNAs.

The experiments follow a logical outline and are conclusive. However, the data presented do not convince in all aspects:

-the pictures in Figure 3A which shall illustrate an increase in alkaline phosphatase activity are not convincing as the increased staining is hard to distinguish from the background.

**Response:** Although changes in alkaline phosphatase activity are not apparent enough to be recognized by naked eyes, optical measurements have shown significant changes from the images shown in the manuscript.

- in Fig 6, the alteration in distribution of actin and annexins is illustrated by single pictures (representative?) but not quantified.

**Response:** Yes, the images are representative, but all samples for each group showed similar distributions in annexins. The outcomes were not quantified because it is difficult to quantify the distributions of annexins and β actin filaments in neurons by an imaging approach.

- it remains unclear whether the proposed CaP deposits form in intact cells or only after disruption of the cell membrane, as indicated in Fig 13. If Ca2+ entry and CaP deposition are a consequence of cell membrane damage, prevention of CaP deposition for treatment as proposed in the discussion would be too late.

**Response**: Multiple factors contribute to cell death. Cell membrane damage is one of these factors, and β actin filament calcification may represent another cell death-inducing factor. Preventing annexin-induced β actin filament calcification may partially alleviate neuronal death. This point is addressed in the revised manuscript (page 19 of the revised manuscript).

The individual data points underlying the summary statistics are not available.

**Response:** All raw data are now available in the Supplemental Information section.
